# Relationship between Salivary Amylase and Xerostomia in Intensity-Modulated Radiation Therapy for Head and Neck Cancer: A Prospective Pilot Study

**DOI:** 10.3390/curroncol29090516

**Published:** 2022-09-15

**Authors:** Francesca De Felice, Maria Giulia Scarabelli, Raffaella De Pietro, Giuseppina Chiarello, Federico Di Giammarco, Carlo Guglielmo Cattaneo, Giuliana Lombardo, Francesca Romana Montinaro, Miriam Tomaciello, Mario Tombolini, Daniela Messineo, Pier Luigi Di Paolo, Claudia Marchetti, Daniela Musio, Vincenzo Tombolini

**Affiliations:** 1 Department of Radiotherapy, Policlinico Umberto I, Sapienza University of Rome, 00161 Rome, Italy; 2 Department of Oral and Maxillo Facial Sciences, Policlinico Umberto I, Sapienza University of Rome, 00161 Rome, Italy; 3 Department of Radiological, Oncological and Pathological Sciences, Policlinico Umberto I, Sapienza University of Rome, 00161 Rome, Italy; 4 Department of Imaging, IRCCS Ospedale Pediatrico Bambino Gesù, 00165 Rome, Italy; 5 Department of Woman and Child Health and Public Health, Fondazione Policlinico Universitario A. Gemelli IRCCS, 00168 Rome, Italy; 6 Department of Life Sciences and Public Health, Università Cattolica del Sacro Cuore, 00168 Rome, Italy

**Keywords:** salivary amylase, xerostomia, parotid, salivary gland, IMRT, radiotherapy, toxicity

## Abstract

Purpose. A single-institution prospective pilot study was conducted to the assess correlation between salivary amylase and xerostomia in patients with head and neck squamous cell carcinoma (HNSCC) treated with intensity-modulated radiotherapy (IMRT). Methods and materials. Serum saliva amylase, clinician-reported xerostomia (using Common Terminology Criteria for Adverse Events), and patient-reported xerostomia (using 8-item self-reported xerostomia-specific questionnaire) were prospectively collected at baseline, during treatment and thereafter. Correlations between variables were assessed by correlation matrices. Results. Twelve patients with locally advanced HNSCC formed the cohort. Eighty-three percent were male, 75% were smokers, 100% had clinical positive lymph nodes at diagnosis, and 42% received induction chemotherapy. All patients received IMRT with concurrent cisplatin-based chemotherapy. No grade ≥4 xerostomia was observed. Severe (G3) acute and late xerostomia occurred in five cases (41.7%) and two cases (16.7%), respectively. Patient-reported xerostomia scores were highly correlated with the clinician-reported scores (ρ = 0.73). A significant correlation was recorded between the concentration of amylase and the acute (ρ = −0.70) and late (ρ = −0.80) xerostomia. Conclusion. Preliminary results are encouraging. Prospective clinical trials are needed to define the value of salivary amylase in the management of HNSCC tumors.

## 1. Introduction

Currently, surgery, radiation therapy (RT), chemotherapy, or combinations of these therapeutic modalities represent the classical options for managing head and neck squamous cell carcinoma (HNSCC) [1,2]. Primary definitive RT is usually performed in the case of surgical approaches related to significant functional loss or in patients with medical contraindication to surgery [1,2]. Due to the anatomic proximity to various structures, the risk of radiation-induced toxicities is important and imposes an adequate assessment, support, and surveillance before, during, and after treatment. Xerostomia represents the most common RT side effect. With the shift from a three-dimensional technique to intensity-modulated technology (IMRT), interpretation of the clinical significance of the xerostomia identified in HNSCC has become a challenge. In the last decades, various clinical studies searching for predictors of radiation-induced xerostomia have been conducted and the vast majority of these trials focused on factors following parotid-sparing irradiation [3,4]. One important consequence is that the spared glands in each patient are expected to produce the majority of saliva after RT. After RT, parotid glands are damaged, thus their secretion is decreased and their secretory components are altered [5]. Salivary amylase is a glucose polymer cleavage enzyme mainly produced by parotid glands and it has a major physiologic role in food digestion. Its level can be easily measured in a blood sample. A rise in serum salivary amylase seems to be strictly related to the quantity of salivary tissue exposed to ionizing radiation [6]. Ionizing radiation induces damage to muscarinic receptors involved in secretary responsiveness, with consequent destruction of serous cells, which are rich in amylase [6].

Our aim is to prospectively explore the existence of a direct relationship between the serum salivary amylase quantitative changes during treatment and development of xerostomia. It may be useful in assessing the degree of radiation-induced damage.

## 2. Materials and Methods

### 2.1. Eligibility Criteria

Consecutive patients with a locally advanced HNSCC treated with either definitive or adjuvant chemoradiotherapy (CRT) were registered on this prospective study. The study was approved by the institutional review board (# 6452). Written informed consents were obtained before enrollment. All cases were discussed in a multidisciplinary head and neck meeting before treatment. Patients with contraindications to radiotherapy or chemotherapy or those with synchronous tumors, or a history of neurological or psychiatric disorders, or previous RT to the head and neck region were considered not eligible for the study. Clinical examinations, including complete medical history and careful physical examination with nasopharyngolaryngoscopy were combined with radiologic imaging, chest computed tomography and diffusion-weighted magnetic resonance imaging of the head and neck region, to assess the precise local (T), regional nodal (N), and distant (M) extent of the tumor. Clinical staging was according to the 8th edition of the American Joint Committee on Cancer staging manual [7]. Preventive dental care and nutritional evaluation was performed before treatment [8].

### 2.2. Treatment

IMRT was applied to all patients with a 6–15 MV photon beam. Firstly, adequate target coverage was achieved and then parotid gland sparing was recommended. Contralateral parotid gland sparing was the main objective of the treatment planning. The irradiated volume of each parotid gland was separately considered.

In case of primary (C)RT, the prescription dose to the gross tumor volume was 67.5 Gy in 2.25 Gy/fraction, whereas a total dose of 60 Gy (2 Gy/fraction) and 54 Gy (1.8 Gy/fraction) was delivered to the high-risk and low-risk clinical target volume, respectively. In case of adjuvant (C)RT, the prescribed dose was 66–60 Gy (2 Gy/fraction) to the tumor bed and 60–50 Gy (2 Gy/fraction) to the subclinical target volumes. Concomitant cisplatin-based chemotherapy (100 mg/m^2^ day 1–21) was administered. Induction chemotherapy was proposed in case of N3 classification or bulky disease and consisted of three cycles of docetaxel (75 mg/m^2^ day 1), cisplatin (75 mg/m^2^ day 1), and 5-fluorouracil (750 mg/m^2^ days 1–5).

### 2.3. Salivary Amylase Evaluation

Each patient blood sample was collected in an ethylenediaminetetraacetic acid (EDTA)-treated tube and then analyzed for serum amylase enzymes. Serum amylase were measured at eleven measurement points: at baseline (t0); during the first week of treatment, before each fraction of CRT (t1, day one; t2, day two; t3, day three; t4, day four; and t5, day five); and then at intervals of one week after the start of CRT (t6, week two; t7, week three; t8, week four; t9, week five; t10, and week six). The delta amylase value was defined as the change between baseline and higher serum amylase value.

### 2.4. Xerostomia Evaluation

Clinician-reported xerostomia and patient-reported xerostomia were recorded. Xerostomia was measured at the same serum amylase time intervals and thereafter during standard follow-up program. Both acute and late xerostomia were chosen as clinical variables. Acute xerostomia was defined as the worst clinician-reported value during treatment; late xerostomia was defined as the worst clinician-reported value during follow-up. Follow-up consisted of physical examination and imaging according to institutional guidelines [9]. The Common Terminology Criteria for Adverse Events (CTCAE) version 5.0 instrument was used for acute and late toxicity clinician reporting. All patients were instructed to complete the 8-item self-reported xerostomia-specific questionnaire (xQ) at every time point [10]. Patients rated each answer on an 11-point ordinal Likert scale from 0 to 10, with higher scores indicating greater xerostomia or discomfort due to xerostomia.

### 2.5. Statistical Analysis

Statistical analysis was performed using R-Studio 0.98.1091 software. Standard descriptive statistics was used to evaluate the distribution of each variable. Continuous variables were reported as means ± standard deviation and categorical variables as frequencies or percentages. Correlations between variables were assessed by correlation matrices and the use of either Pearson (r; parametric) or Spearman (ρ; non-parametric) correlation methods where appropriate. The Kruskal–Wallis test was used to compute if there was any significant difference between the average values of amylase in the time point. A *p*-value < 0.05 was considered significant.

## 3. Results

From January 2019 to December 2020, a total of 12 adult patients with histologically confirmed HNSCC who were scheduled for CRT at our institution were prospectively enrolled. Baseline characteristics are listed in Table 1. Median age of the patient group was 58.5 (±8.5) years. Almost all were male (n = 10; 83.3%) and most patients had been exposed to the classical risk factors: the majority (n = 9; 75%) was former or current tobacco smokers with a tobacco exposure > 10 pack/years in 7 cases. Half of the patients (n = 6; 50%) had oropharyngeal cancer. Five patients (41.6%) had N3 or bulky disease at diagnosis and received induction chemotherapy. All patients complete their planned CRT scheme reaching a cumulative dose of 200 mg/m^2^ cisplatin. The median follow-up times since the completion of CRT was 28 months (range 15–40).

All patients received comprehensive bilateral neck irradiation. The mean doses to the ipsilateral parotid gland, the contralateral parotid gland, the ipsilateral parotid irradiated volume (IPIV), and contralateral parotid irradiated volume (CPIV) are detailed in Table 2. The contralateral parotid gland received on average a mean dose of 24.2 Gy.

**Figure 1:**
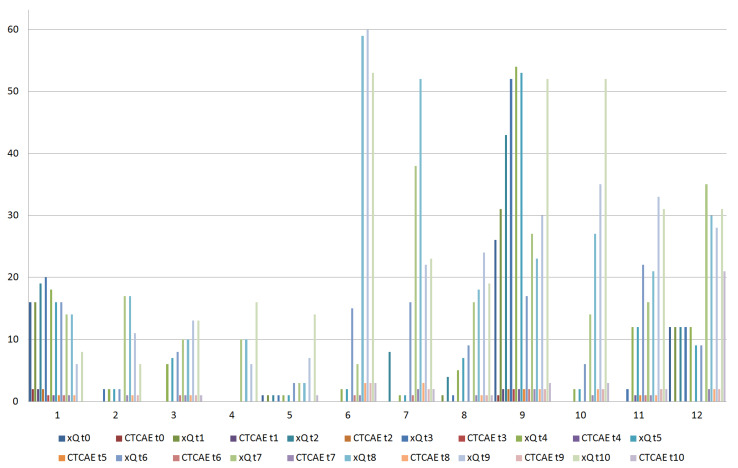
Patient-reported (xQ) and clinician-reported (CTCAE) acute xerostomia results. Time points: at baseline (t0); during the first week of treatment, before each fraction of CRT (t1, day one; t2, day two; t3, day three; t4, day four; t5, day five); and then at intervals of one week after the start of CRT (t6, week two; t7, week three; t8, week four; t9, week five; t10, week six).

Figure 1 provided both patient-reported and clinician-reported acute xerostomia results. A general worsening in xerostomia over time was recorded. Severe acute xerostomia occurred in five cases (41.7%). Among these patients, severe xerostomia persisted in two cases (16.7%) late after treatment. The xQ summary scores were highly correlated with the clinician-reported scores (ρ = 0.73).

**Figure 2:**
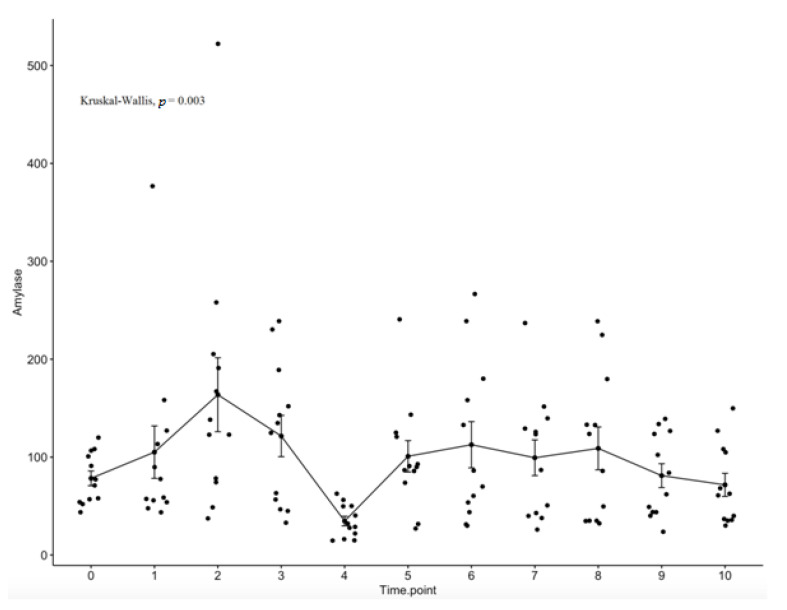
The simple effect of time for serum salivary amylase. Time points: at baseline (t0); during the first week of treatment, before each fraction of CRT (t1, day one; t2, day two; t3, day three; t4, day four; t5, day five); and then at intervals of one week after the start of CRT (t6, week two; t7, week three; t8, week four; t9, week five; t10, week six).

Serum salivary amylase values as a function of time are depicted in Figure 2. The main effect of the time showed significant difference for amylase with a significant decrease in the levels of salivary amylase from day three (t3) of radiotherapy to day four (t4) of RT (*p* = 0.003).

**Figure 3:**
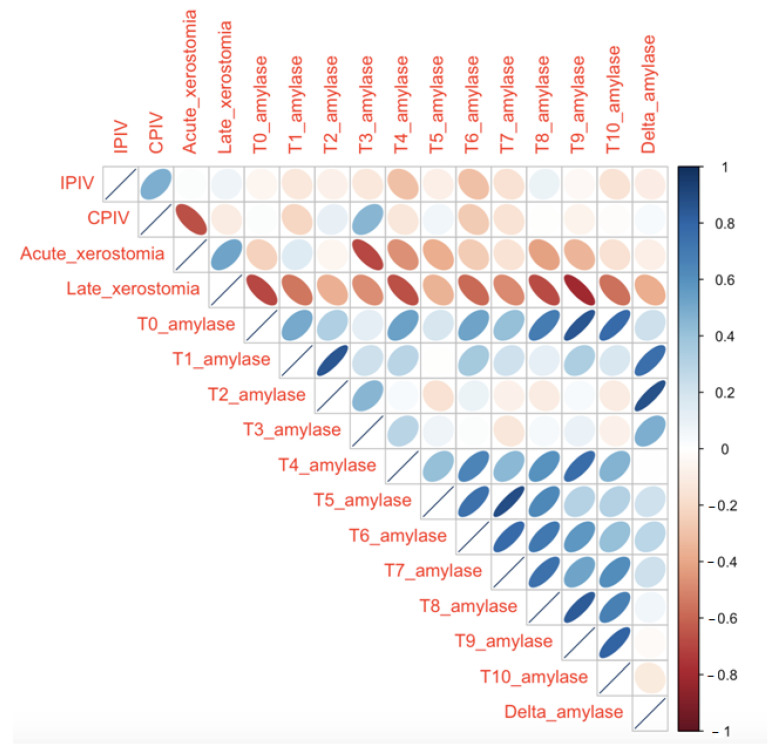
Correlation matrix between different clinical variables. Each ellipse in the table represents a correlation between the two variables. The diagonal indicates that each variable perfectly correlates with itself. Time points: at baseline (t0); during the first week of treatment, before each fraction of CRT (t1, day one; t2, day two; t3, day three; t4, day four; t5, day five); and then at intervals of one week after the start of CRT (t6, week two; t7, week three; t8, week four; t9, week five; t10, week six). Delta amylase was defined as the change between baseline and higher serum amylase value.

A correlation matrix was used to summarize data. The coefficients between the different clinical variables are reported in Figure 3. A significant correlation was obtained between the concentration of amylase and the acute and late xerostomia. Late xerostomia negatively correlated with amylase, especially at t9 (ρ = −0.80). Considering correlation between amylase and acute xerostomia, the main statistical significance was recorded at t3 (ρ = −0.70). CPIV negatively correlated with acute xerostomia (ρ = −0.66), whereas no significant correlation between irradiated parotid volume, both IPIV and CPIV, and amylase values was noted.

## 4. Discussion

This prospective clinical study of patients with HNSCC treated with CRT suggested a relationship between rapid acute increase and subsequent decrease in serum salivary amylase and xerostomia.

Our study showed that the simple effect of the time for amylase was statistically significant, especially between day three and day four of RT (*p* = 0.003). Interestingly, both acute and late xerostomia negatively correlated with salivary amylase. The more loss of serous salivary cells and their atrophy structure there is, the less ability to produce amylase there is, consecutively increasing xerostomia severity. These clinical results were consistent with prior studies. The information obtained from the published studies is limited and most of the available references are old, which might mean the topic is not frontier research [11]. However, we believe that serum salivary amylase activity is valuable to a certain extent to clinical measure. The relationship between serum salivary amylase level and xerostomia is scarcely reported in the scientific literature [6,12,13,14]. In the past two decades, several clinical trials have been performed to determine the role of salivary amylases in predicting xerostomia. According to our results, these studies reported a transient hyperamylasemia after a dose up to 4 Gy to the head and neck region. After that, serum amylase declined to normal over a further 24–48 h.

One unexpected finding in our study is that CPIV negatively correlated with acute xerostomia severity. This finding may reflect the small sample size in this cohort, or can be explained by the multicollinearity phenomenon. In fact, multiple factors likely contributed to the development of xerostomia in patients undergoing CRT for HNSCC. The salivary gland system includes three paired major glands (parotid, submandibular, and sublingual) and approximately 1000 minor salivary glands spread throughout the pharynx (from the skull base—tubarial glands [15]) and the oral cavity submucosa [8]. This anatomical distribution complicates measuring a difference in toxic effects caused by irradiation to different glands. Since these glands are situated closely together, they often receive a comparable dose during treatment. Therefore, despite a parotid glands-sparing strategy, the toxic effect caused by dose to different salivary glands may be responsible for the development of xerostomia.

While this study was specifically designed to evaluate a relationship between salivary amylase and xerostomia, it should be noted that parotid glands continued to recover their function over 24 months. Only two patients had severe xerostomia at the last follow-up. This finding agrees with the fact that long term xerostomia can improve over time, and even longer follow-up with detailed longitudinal reporting and dosimetric correlations will be of interest. To our knowledge, this is the first report of such data. The strengths of this study were its prospective design with salivary amylase and patient-reported levels of xerostomia collected on a regular basis and in a consistent fashion during the entire period of the study. However, the inherent limitations of a single-institution series of modest power apply here. Our results are preliminary, and the sample size was insufficient for adjustment for uncontrolled biases, such as induction chemotherapy and primary tumor site, thus should be interpreted with caution. In addition, several outcomes of interest such as treatment response, survival, and recurrence rates were not analyzed because of the low number of patients and the relatively short follow-up time.

Serum salivary amylase variations have an unknown impact on xerostomia, and maybe rigorous serum salivary amylase and xerostomia reporting can be useful to capture these data. An important need exists for a clinical support tool that distills this information into an easily interpretable method that is available to clinicians of all HNSCC knowledge levels and at all centers. Such a support tool would help clinicians to interpret xerostomia grade and enable them to support optimal treatment decisions for each HNSCC patient. Serum salivary amylase can probably be used as a biological indicator to xerostomia management. One major unanswered question relates to the definition of a cut-off value for amylase as a potential predictive factor of xerostomia. Our study did not provide insight into this issue, but rather suggested that differences in amylase value over time can be detected in HNSCC patient population treated with CRT. Salivary amylase seems to be a reasonable composite study endpoint that can be meaningful both for clinicians and patients. This finding would need to be verified in a large cohort to obtain a definite cut-off value to conclusively address this issue. Comprehensive scientific research that includes new clinical measures (i.e., using serum salivary amylase as a biomarker) is warranted to ameliorate the therapeutic index of RT in HNSCC patients.

## 5. Conclusions

Irradiation of salivary glands determined a changing trend with an initial increase from day 1 to day 2 followed by a significant decrease from day 3 to day 4 of RT. Our results indicated that for locally advanced HNSCC, salivary amylase was associated with xerostomia over time. These findings will help to guide future research on biomarkers and patient-tailored treatment protocols in HNSCC management.

## Figures and Tables

**Table 1:** Baseline characteristics.

Characteristic	Patient (%)
Age (years)	58.5
median (SD)	8.5
Gender	
male	10 (83.3)
female	2 (16.7)
Smoker	
never	3 (25.0)
former	2 (16.7)
current	7 (58.3)
≤10 pack/years	2 (16.7)
>10 pack/years	7 (58.3)
Alcohol consumption	
never	6 (50.0)
former	1 (8.3)
current	5 (41.7)
≤21 units/week	5 (41.7)
>21 units/week	1 (8.3)
Tumor localization	
oropharynx	6 (50.0)
HPV-positive	2 (33.3)
HPV-negative	4 (66.7)
larynx	2 (16.7)
nasopharynx	2 (16.7)
hypopharynx	1 (8.3)
oral cavity	1 (8.3)
Clinical tumor stage (cT)	
2	6 (50.0)
3	2 (16.7)
4a	4 (33.3)
Clinical node stage (cN)	
1	3 (25.0)
2	7 (58.3)
3	2 (16.7)
Treatment	
definitive CRT	11 (91.7)
adjuvant CRT	1 (8.3)

SD: standard deviation; CRT: chemoradiotherapy.

**Table 2:** Parotid glands: mean doses and irradiated volume.

	Mean Dose (Gy)	Irradiated Volume (cc)
Ipsilateral parotid gland	30.0 (±5.7)	0.9 (±1.1)
Contralateral parotid gland	24.2 (±3.3)	0.6 (±0.8)

Gy: Gray; cc: cubic centimeter.

## Data Availability

Data are available from the authors upon reasonable request.
